# Comparative Diagnostic Accuracy of Fine-Needle Aspiration Cytology Versus Fine-Needle Non-aspiration Cytology in Thyroid Lesions: A Cross-Sectional Study

**DOI:** 10.7759/cureus.112727

**Published:** 2026-07-15

**Authors:** Vanniyar Selvan M, Manoj K Paswan, Saurav Banerjee, Prachi Hansdah, Anshu Jamaiyar

**Affiliations:** 1 Pathology, Rajendra Institute of Medical Sciences, Ranchi, IND

**Keywords:** cytodiagnosis, fine-needle aspiration, fine-needle non-aspiration, thyroid neoplasms, thyroid nodule

## Abstract

Background

Fine-needle aspiration cytology (FNAC) and fine-needle non-aspiration cytology (FNNAC) are commonly used techniques for the evaluation of thyroid lesions. Differences in smear quality, cellular preservation, and diagnostic performance between these methods remain a subject of interest. Our study compared FNAC and FNNAC in thyroid lesions using histopathological examination as the reference standard.

Methodology

A cross-sectional observational study was conducted among 110 patients with clinically palpable thyroid swellings, of whom 75 patients who underwent thyroid surgery with available histopathological examination were included in the final analysis. Both FNAC and FNNAC were performed on the same lesion during the same sitting. Smears were assessed for adequacy, cellular preservation, and diagnostic categorization using the Bethesda System for Reporting Thyroid Cytopathology. Histopathological examination served as the reference standard for cytohistological correlation and assessment of diagnostic performance.

Results

The mean age of participants was 42.88 ± 13.59 years. Superior smear quality was observed in 55 (73.3%) FNAC samples and 59 (78.7%) FNNAC samples. Bethesda Category II lesions constituted the majority of cases with both techniques. FNNAC reduced the proportion of indeterminate lesions and identified a higher proportion of malignant lesions. Histopathology revealed 53 (70.7%) benign and 22 (29.3%) malignant lesions. FNNAC demonstrated a stronger correlation with histopathological diagnosis and showed better preservation of cellular architecture with less blood contamination. Diagnostic accuracy was numerically higher with FNNAC (63, 84.0%) than with FNAC (58, 77.3%), with corresponding sensitivities of 11 (50.0%) and 9 (40.9%), respectively; however, the difference in diagnostic accuracy was not statistically significant (McNemar’s test, p = 0.228).

Conclusions

Both FNAC and FNNAC are effective techniques for the cytological evaluation of thyroid lesions. FNNAC demonstrated higher smear quality, improved cellular preservation, higher specificity, and numerically higher overall diagnostic accuracy than FNAC. Although the difference in diagnostic accuracy was not statistically significant, FNNAC may serve as a valuable alternative or complementary technique for improving smear quality in thyroid cytology.

## Introduction

Thyroid nodules are one of the most common endocrine disorders seen in clinical practice, and they become more common with increasing age, being more common in women, associated with iodine deficiency, and the widespread use of high-resolution imaging modalities [1,2]. Although the majority of thyroid lesions are benign, a small but clinically significant proportion harbor malignancy, necessitating accurate preoperative evaluation to guide appropriate management and avoid unnecessary surgical intervention [1,2]. Early differentiation between neoplastic and non-neoplastic lesions remains essential because delayed diagnosis of malignant thyroid lesions may adversely affect prognosis, while overdiagnosis may lead to avoidable thyroidectomy and associated morbidity [3]. Fine-needle aspiration cytology (FNAC) has long been regarded as the primary minimally invasive diagnostic modality for evaluation of thyroid swellings because of its simplicity, cost-effectiveness, rapid turnaround time, and high diagnostic accuracy [4,5]. Despite its established utility, conventional FNAC is frequently limited by blood contamination, clotting artifacts, and cellular trauma caused by negative suction pressure, which may compromise smear adequacy and cytomorphological interpretation, particularly in highly vascular thyroid lesions [6,7].

To overcome these limitations, fine-needle non-aspiration cytology (FNNAC), also referred to as capillary sampling cytology, has been introduced as an alternative technique in which cellular material is obtained through capillary action without application of suction pressure [7,8]. Comparisons of FNAC and FNNAC in the diagnosis of thyroid lesions have shown mixed results in several studies [8,9]. There have been many studies evaluating the usefulness of FNAC and FNNAC, but it is not clear which technique is better than the other [8,10]. It is also not clear whether the relative performance of FNAC and FNNAC is the same for all lesion types, regarding their consistency, vascularity, and operator’s experience [11].

Limited studies incorporating standardized smear scoring systems and histopathological correlation are available from tertiary care centers in the Indian subcontinent [8]. Therefore, comparative evaluation using objective adequacy criteria and cytohistological concordance remains clinically relevant [8]. In view of these considerations, the present study was undertaken to compare FNAC and FNNAC in thyroid lesions with respect to smear adequacy, cytomorphological quality, and diagnostic performance using histopathological examination as the reference standard. The study also aimed to assess the utility of both techniques within the framework of the Bethesda System for Reporting Thyroid Cytopathology.

## Materials and methods

This cross-sectional observational study was conducted in the Department of Pathology, Rajendra Institute of Medical Sciences (RIMS), Ranchi, over 18 months from July 2024 to January 2026. We evaluated patients presenting with clinically palpable thyroid swellings who were referred for cytological examination. Patients aged more than 14 years with clinically palpable thyroid lesions were included. We excluded patients unwilling to participate, those with bleeding diathesis or coagulopathy, inadequately fixed cytological smears, and cases with insufficient tissue for histopathological correlation. A convenient sampling method was used for participant recruitment. In India, around 2.9% people reported experiencing a thyroid disorder, according to the data from the fifth National Family Health Survey (NFHS-5) [12]. The minimum calculated sample size was 45 with a margin of error of 5%. To enhance the statistical validity and to make a better comparison of the two cytological techniques, 75 patients were included in the final analysis.

During the study period, 110 consecutive patients with clinically palpable thyroid swellings underwent both FNAC and FNNAC. Surgical management was determined independently by the treating surgical team based on standard clinical indications. Histopathological examination was available for 75 surgically excised lesions, and these patients constituted the study population for analysis. FNAC and FNNAC were performed in the same sitting on the same thyroid lesion in each patient.

**Figure 1:**
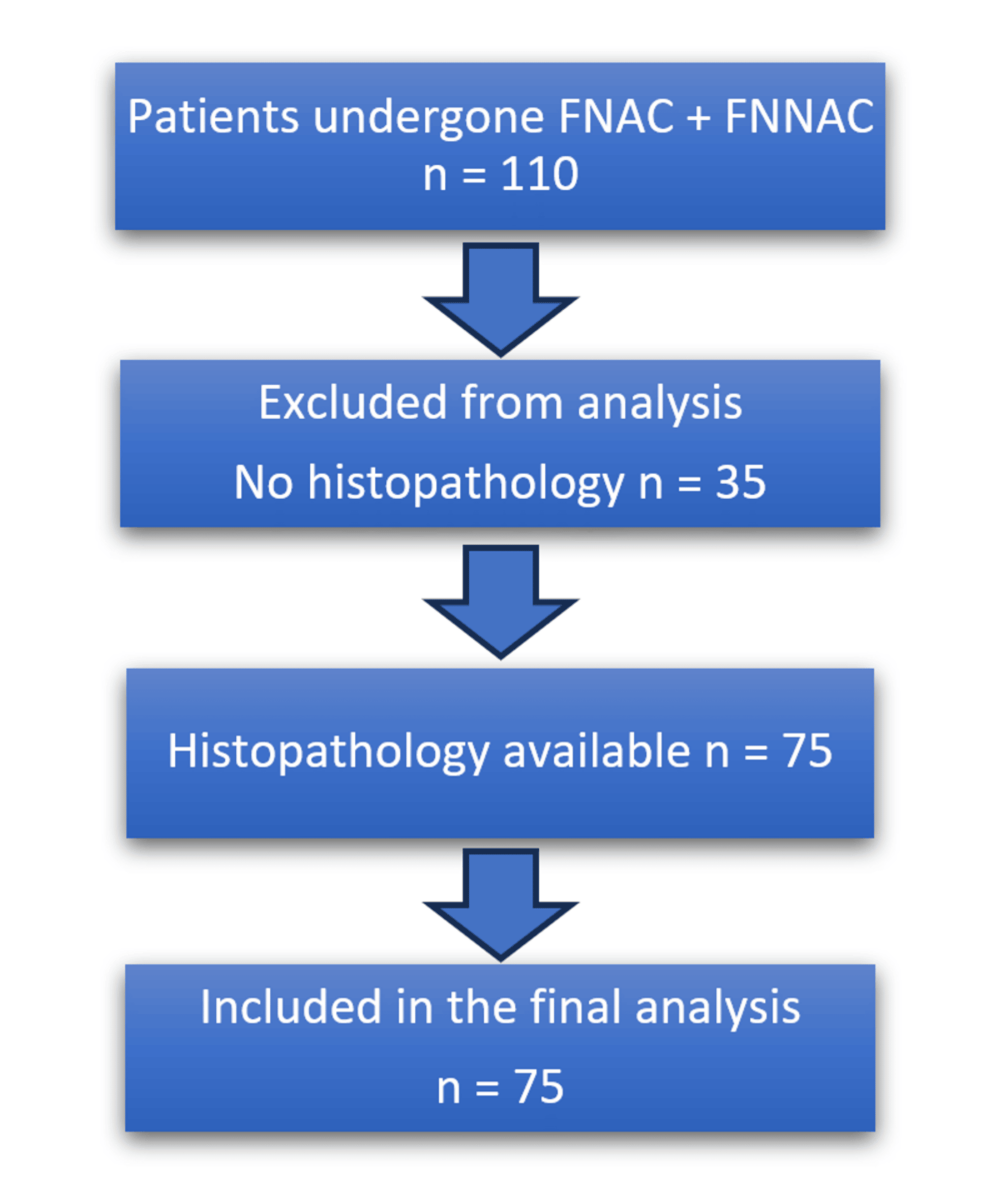
Flow diagram of patient selection and inclusion in the study. FNAC = fine-needle aspiration cytology; FNNAC = fine-needle non-aspiration cytology

FNNAC was performed before FNAC in all patients to minimize aspiration-induced hemorrhage and tissue disruption. Although the sampling sequence was not randomized, both cytological smears were evaluated independently by blinded cytopathologists unaware of the corresponding smear results. Histopathological examination was also performed by a pathologist blinded to the cytological diagnosis. The needle used by FNNAC is a 23-gauge needle that is not used with suction, and multiple passes are made in various directions with a small amount of cellular material drawn in by capillary action [11]. Afterwards, FNAC was performed through the use of a 23-gauge needle mounted on a 10 mL syringe with negative pressure applied during sampling.

The material obtained was smeared on clean glass slides. The samples were air-dried and stained with Leishman-Giemsa stain, and the wet-fixed smears in 95% ethanol were stained with hematoxylin and eosin stain and the Papanicolaou technique. The adequacy, cellularity, and background blood contamination were assessed, as were the presence of architectural preservation and cellular degeneration, in cytological smears. The 2023 Bethesda System for Reporting Thyroid Cytopathology was used to classify thyroid lesions [13]. Histopathological findings of surgical specimens available were used as the gold standard for diagnostic correlation.

Data were entered into Microsoft Excel (Microsoft Corp., Redmond, WA, USA) and analyzed using SPSS software (IBM Corp., Armonk, NY, USA). Descriptive statistics were expressed as mean ± standard deviation and frequency in n (%). The chi-square test was used to assess associations between categorical variables, while agreement between FNAC and FNNAC was evaluated using kappa statistics. A p-value <0.05 was considered statistically significant.

The Institutional Ethics Committee (IEC) provided study approval (approval number: 132 dated 08/02/2025 (officially approved February 2024 in the older format of the IEC certificate)). All participants provided written informed consent before joining the study.

## Results

Among the 110 patients who underwent both FNAC and FNNAC, 75 patients who subsequently underwent thyroid surgery with available histopathological examination were included in the final analysis. The mean age of the study population was 42.88 ± 13.59 years, with 41 (54.7%) patients aged >40 years. Male patients constituted 44 (58.7%) cases. On smear adequacy assessment, FNAC produced superior smears in 55 (73.3%) cases, whereas FNNAC yielded superior smears in 59 (78.7%) cases. Unsuitable smears accounted for 3 (4.0%) FNAC samples and 4 (5.3%) FNNAC samples (Table 1).

**Table 1:** Baseline characteristics and smear adequacy among study participants (N = 75). FNAC = fine-needle aspiration cytology; FNNAC = fine-needle non-aspiration cytology

Variable	n (%)/Mean ± SD
Age (years)	42.88 ± 13.59
18–40 years	34 (45.3%)
>40 years	41 (54.7%)
Sex	
Male	44 (58.7%)
Female	31 (41.3%)
FNAC adequacy	
Superior	55 (73.3%)
Adequate	17 (22.7%)
Unsuitable	3 (4.0%)
FNNAC adequacy	
Superior	59 (78.7%)
Adequate	12 (16.0%)
Unsuitable	4 (5.3%)

Bethesda Category II lesions constituted the majority with both techniques, accounting for 42 (56.0%) FNAC smears and 47 (62.7%) FNNAC smears. We observed a reduction in indeterminate lesions categorized under Bethesda Category III with FNNAC (3 (4.0%)) compared with FNAC (8 (10.7%)). Bethesda Category VI lesions were identified more frequently with FNNAC (11 (14.7%)) than FNAC (7 (9.3%)), indicating improved cytomorphological characterization with the non-aspiration technique (Table 2).

**Table 2:** Comparison of Bethesda categories between FNAC and FNNAC. AUS = atypia of undetermined significance; FLUS = follicular lesion of undetermined significance; FNAC = fine-needle aspiration cytology; FNNAC = fine-needle non-aspiration cytology

Bethesda category	FNAC, n (%)	FNNAC, n (%)
I (non-diagnostic)	6 (8.0%)	6 (8.0%)
II (benign)	42 (56.0%)	47 (62.7%)
III (AUS/FLUS)	8 (10.7%)	3 (4.0%)
IV (follicular neoplasm)	7 (9.3%)	7 (9.3%)
V (suspicious for malignancy)	5 (6.7%)	1 (1.3%)
VI (malignant)	7 (9.3%)	11 (14.7%)

Histopathological examination identified 53 (70.7%) benign lesions and 22 (29.3%) malignant lesions. Comparative analysis demonstrated a statistically significant association between FNAC and FNNAC smear adequacy (χ² = 28.900, p < 0.001), with fair agreement on kappa analysis (κ = 0.203, p = 0.035). We observed that FNNAC upgraded 24 of 34 (70.6%) FNAC smears previously categorized as adequate into superior-quality smears, suggesting improved specimen quality with the non-aspiration technique.

Correlation with histopathological diagnosis showed that FNAC correctly identified 49 of 53 (92.5%) benign lesions, whereas 13 of 22 (59.1%) malignant lesions were misclassified as benign. In comparison, FNNAC correctly identified 52 of 53 (98.1%) benign lesions and demonstrated improved malignant lesion detection, correctly diagnosing 11 of 22 (50.0%) malignant cases. Statistical analysis demonstrated a stronger association between FNNAC and histopathology (χ² = 26.777, p < 0.001) compared with FNAC (χ² = 12.076, p = 0.001) (Table 3).

**Table 3:** Correlation of cytological diagnosis with histopathological examination. FNAC = fine-needle aspiration cytology; FNNAC = fine-needle non-aspiration cytology

Technique	Histopathology	Cytology benign, n (%)	Cytology malignant, n (%)	Chi-square	P-value
FNAC	Benign (n = 53)	49 (92.5%)	4 (7.5%)	12.076	0.001
	Malignant (n = 22)	13 (59.1%)	9 (40.9%)		
FNNAC	Benign (n = 53)	52 (98.1%)	1 (1.9%)	26.777	<0.001
	Malignant (n = 22)	11 (50.0%)	11 (50.0%)		

Diagnostic performance analysis showed that FNAC had a sensitivity of 9 (40.9%), a specificity of 49 (92.5%), a positive predictive value of 9 (69.2%), a negative predictive value of 49 (79.0%), and an overall diagnostic accuracy of 58 (77.3%). FNNAC demonstrated comparatively superior performance with a sensitivity of 11 (50.0%), a specificity of 52 (98.1%), a positive predictive value of 11 (91.7%), a negative predictive value of 52 (82.5%), and an overall diagnostic accuracy of 63 (84.0%) (Table 4).

**Table 4:** Diagnostic performance of FNAC and FNNAC using histopathology as the reference standard. FNAC = fine-needle aspiration cytology; FNNAC = fine-needle non-aspiration cytology; CI = confidence interval

Parameter	FNAC, n (%)	95% CI	FNNAC, n (%)	95% CI
Sensitivity	9 (40.9%)	23.3–61.3%	11 (50.0%)	30.7–69.3%
Specificity	49 (92.5%)	82.1–97.0%	52 (98.1%)	90.1–99.7%
Positive predictive value	9 (69.2%)	42.4–87.3%	11 (91.7%)	64.6–98.5%
Negative predictive value	49 (79.0%)	67.4–87.3%	52 (82.5%)	71.4–90.0%
Diagnostic accuracy	58 (77.3%)	66.7–85.3%	63 (84.0%)	74.1–90.6%

Although FNNAC correctly diagnosed eight cases that FNAC missed and FNAC correctly diagnosed only three cases missed by FNNAC, this difference did not reach statistical significance (McNemar’s test, p = 0.228) (Table 5).

**Table 5:** Paired comparison of the diagnostic accuracy of FNAC and FNNAC using McNemar’s test. McNemar’s test: χ² = 1.455, p = 0.228. FNAC = fine-needle aspiration cytology; FNNAC = fine-needle non-aspiration cytology

Paired analysis	FNNAC correct	FNNAC incorrect	Total
FNAC correct	55	3	58
FNAC incorrect	8	9	17
Total	63	12	75

## Discussion

Fine-needle cytology remains the preferred minimally invasive investigation for preoperative evaluation of thyroid lesions because of its simplicity, rapidity, and high diagnostic utility [4,5]. Both FNAC and FNNAC demonstrated good diagnostic utility. FNNAC produced higher-quality smears and numerically higher diagnostic accuracy than FNAC, although the difference in overall diagnostic accuracy was not statistically significant on paired comparison. Compared to previous studies, which reported a female predominance [13,14], a male predominance (44 (58.7%)) was observed in our study. A female predominance (65%-80%) was reported by Maurya et al. and Sharma et al., which could be attributed to differences in the institutional demographic, referral pattern, and relatively small sample size rather than the underlying disease epidemiology [15,16]. Smear adequacy is a critical determinant of cytological interpretation [8,17]. We observed superior smears in 59 (78.7%) FNNAC cases compared with 55 (73.3%) FNAC cases. Additionally, FNNAC upgraded 24 of 34 (70.6%) FNAC smears previously categorized as adequate into superior-quality smears, with a significant association between the two techniques (χ² = 28.900, p < 0.001). These findings are concordant with those of Sharma et al. and Sasikumar et al., who demonstrated reduced blood contamination and better preservation of follicular architecture with FNNAC [16,17]. Song et al. also emphasized that capillary sampling produces cleaner backgrounds with improved cellular preservation [8]. The improved smear quality observed with FNNAC in our study may be attributed to reduced vascular trauma and minimal hemodilution caused by the absence of suction pressure [8,16]. The Bethesda System for Reporting Thyroid Cytopathology has further standardized and rendered the interpretation of thyroid cytology more reproducible, with the introduction of a uniform set of diagnostic categories associated with the risk of malignancy and treatment recommendations [6]. Although the usefulness of conventional FNAC is well established, it is often hindered by blood contamination and blood clotting artifacts, which may affect smear quality and cytomorphological interpretation, especially in highly vascular thyroid lesions [7].

The majority of the lesions with both techniques were Bethesda category II (42 (56.0%) FNAC smears or 47 (62.7%) FNNAC smears). We also found that the number of Bethesda Category III lesions was reduced from 8 (10.7%) in FNAC to 3 (4.0%) in FNNAC, suggesting greater interpretive clarity with non-aspiration sampling. Kumar and Yaam and Shah et al. have reported similar observations with FNNAC showing superior cytomorphological preservation [18,19].

Histopathological correlation is still considered the gold standard for the assessment of diagnostic accuracy [9-11]. FNAC had a sensitivity of 9 (40.9%), a specificity of 49 (92.5%), and an overall diagnostic accuracy of 58 (77.3%), while FNNAC had a sensitivity of 11 (50.0%), a specificity of 52 (98.1%), and a diagnostic accuracy of 63 (84.0%). Better concordance of FNNAC with the gold standard is further substantiated by the higher value of the chi-square statistic (χ² = 26.777, p < 0.001) compared to FNAC (χ² = 12.076, p = 0.001). Song et al. and Wang et al. also found cleaner smears, less blood contamination, and fewer repeat aspirations with FNNAC [8]. FNAC, however, remained useful for dense or fibrotic masses where suction may help in obtaining sufficient material [8,15]. FNNAC demonstrated numerically higher diagnostic accuracy than FNAC (84.0% vs. 77.3%); however, the difference was not statistically significant on paired comparison (McNemar’s test). Consequently, the relatively low sensitivity observed in our study may be attributable to the inclusion of diagnostically challenging lesions, particularly follicular-patterned neoplasms, the analysis being restricted to surgically excised cases with histopathological confirmation, and the use of strict Bethesda-based categorization. These factors may have increased false-negative results and contributed to lower sensitivity.

The strengths of our study include direct comparison of FNAC and FNNAC in the same patients, histopathological confirmation as the reference standard, standardized Bethesda-based reporting, blinded interpretation of cytology and histopathology, and paired statistical analysis using McNemar’s test. However, several limitations should be acknowledged. Only surgically managed patients with available histopathology were included, introducing potential verification bias and limiting the generalizability of the findings to patients managed conservatively. The single-center design, relatively small number of malignant cases, and operator dependence may also have influenced the results. Furthermore, FNNAC was consistently performed before FNAC; although this approach minimized aspiration-induced hemorrhage, the non-randomized sampling sequence may have introduced order-related bias.

## Conclusions

Both FNAC and FNNAC proved to be reliable methods for the cytological evaluation of thyroid lesions. FNNAC produced higher smear quality, better cellular preservation, less blood contamination, and numerically higher specificity and overall diagnostic accuracy than conventional FNAC. Although the difference in diagnostic accuracy between the two techniques was not statistically significant on paired comparison, FNNAC demonstrated improved specimen quality and excellent concordance with histopathology. These findings suggest that FNNAC is a useful alternative or complementary technique to conventional FNAC for the evaluation of thyroid nodules, particularly where improved smear quality and cytomorphological preservation are desired. Further multicenter studies with larger sample sizes are needed to validate these findings.

## Appendices

**Table 6:** Contingency table for FNAC compared with histopathological diagnosis. FNAC = fine-needle aspiration cytology

FNAC	Histopathology malignant	Histopathology benign	Total
Malignant	9 (true positive)	4 (false positive)	13
Benign	13 (false negative)	49 (true negative)	62
Total	22	53	75

**Table 7:** Contingency table for FNNAC compared with histopathological diagnosis. FNNAC = fine-needle non-aspiration cytology

FNNAC	Histopathology malignant	Histopathology benign	Total
Malignant	11 (true positive)	1 (false positive)	12
Benign	11 (false negative)	52 (true negative)	63
Total	22	53	75
